# The genome sequence of the Emperor moth, *Saturnia pavonia *(Linnaeus, 1758)

**DOI:** 10.12688/wellcomeopenres.20652.1

**Published:** 2024-02-19

**Authors:** Liam M. Crowley, Ellen Baker, Peter W. H. Holland, University of Oxford and Wytham Woods Genome Acquisition Lab, Darwin Tree of Life Barcoding collective, Wellcome Sanger Institute Tree of Life Management, Samples and Laboratory team, Wellcome Sanger Institute Scientific Operations: Sequencing Operations, Wellcome Sanger Institute Tree of Life Core Informatics team, Tree of Life Core Informatics collective, Darwin Tree of Life Consortium

**Affiliations:** 1 University of Oxford, Oxford, England, UK

**Keywords:** Saturnia pavonia, Emperor moth, genome sequence, chromosomal, Lepidoptera

## Abstract

We present a genome assembly from an individual male *Saturnia pavonia* (the Emperor moth; Arthropoda; Insecta; Lepidoptera; Saturniidae). The genome sequence is 489.9 megabases in span. Most of the assembly is scaffolded into 30 chromosomal pseudomolecules, including the Z sex chromosome. The mitochondrial genome has also been assembled and is 15.29 kilobases in length. Gene annotation of this assembly on Ensembl identified 11,903 protein coding genes.

## Species taxonomy

Eukaryota; Metazoa; Eumetazoa; Bilateria; Protostomia; Ecdysozoa; Panarthropoda; Arthropoda; Mandibulata; Pancrustacea; Hexapoda; Insecta; Dicondylia; Pterygota; Neoptera; Endopterygota; Amphiesmenoptera; Lepidoptera; Glossata; Neolepidoptera; Heteroneura; Ditrysia; Obtectomera; Bombycoidea; Saturniidae; Saturniinae; Saturniini; *Saturnia*; *Eudia*; *Saturnia pavonia* (Linnaeus, 1758) (NCBI:txid332931).

## Background

The family Saturniidae, named after the ringed planet Saturn, includes over 2000 species of moth worldwide and includes emperor moths and giant silk moths. Two species of Saturniidae have been recorded in Britain: *Saturnia pavonia* (the Emperor moth or Small Emperor moth, a resident breeding species) and *S. pyri* (the Giant peacock moth, the few records likely to be escapees from captive breeding). As befits the family and genus name, *S. pavonia* has large eyespots in the centre of all four wings, each with a black centre surrounded by a buff yellow ring and arcs formed from blue or red scales. The four coloured eyespots give the moth a striking appearance, especially as the moth is one of the largest in Britain or Northern Europe with a wingspan of 60 mm in males or 80 mm in females. Ford (1967) speculated that a hypothesised role of the eye-spots in predator deterrence could be accentuated immediately after eclosion from the pupa, when the wings are still puckered.

The Emperor moth *S. pavonia* is found widely across Europe, from the north of Finland and Norway to the south of Spain and Italy. The range also extends further east in Eurasia to Russia, Kazakhstan and Mongolia ( GBIF Secretariat, 2023). In Britain and Ireland, the moth has been recorded from the south coast of England to the north of Scotland, but is commonest on acid heathlands, moorlands and coastal sand dunes ( NBN Atlas Partnership, 2023).

Adult males are day-flying and particularly active on bright warm days in April and May, when they may be seen flying in search of scent trails released by receptive females. Much has been written about the potency of the *S. pavonia* female sex pheromones and the distances over which males detect females, but many estimates are based conjecture rather than controlled experimentation. After mating, eggs are laid on the foodplant and larvae develop rapidly though summer. On heathlands, the preferred larval foodplant is heather, *Calluna vulgaris*, but the larvae will also eat leaves of hawthorn, apple, bramble and many other shrubs and trees ( South, 1961). Late instar larvae are bright green with black hoops studded with yellow globular outgrowths (scoli); a genetic variant with pink-coloured scoli has also been reported ( Laussmann *et al.*, 2012). When physically stimulated, hollow spines on the scoli secrete a blend of proteins and aromatic compounds thought to deter pathogens or predators; benzonitrile is the dominant small molecule but the secreted proteins have not been identified ( Deml & Dettner, 1990; Laussmann *et al.*, 2012). Larvae pupate inside a tough silken cocoon attached to stems and twigs of the foodplant near the ground. The species overwinters at the pupal stage, sometimes for two or more winters ( Laussmann *et al.*, 2012).

Here we report a complete genome sequence for the Emperor moth *Saturnia pavonia* determined as part of the Darwin Tree of Life project. The genome sequence of *S. pavonia* will facilitate research into the biochemical basis of chemical defence and pheromone communication in insects, and contribute to the growing set of resources for studying molecular evolution in the Lepidoptera.

## Genome sequence report

**Figure 1.**
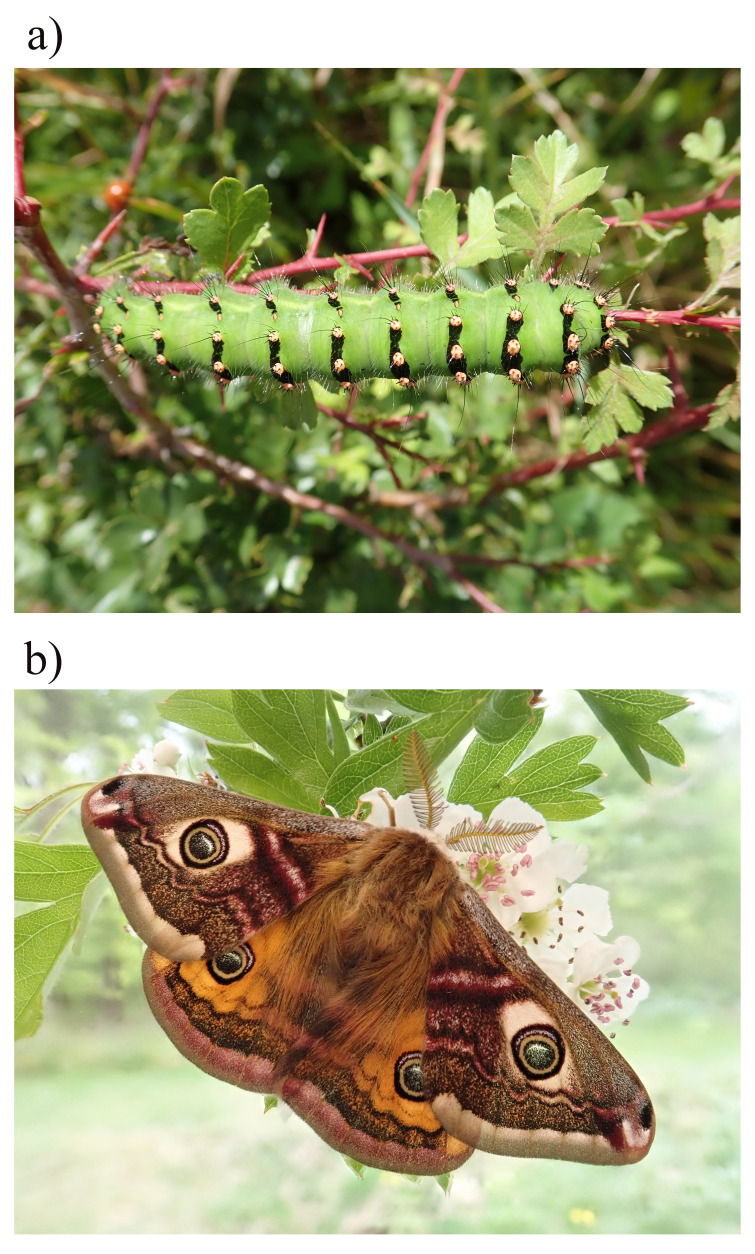
Photograph of the *Saturnia pavonia* (ilSatPavo1) specimen used for genome sequencing; **a**) Larva, **b**) Adult.

The genome was sequenced from one male *Saturnia pavonia* ( Figure 1) reared from a larva collected in Wytham Woods, Oxfordshire, UK (51.77, –1.33). A total of 50-fold coverage in Pacific Biosciences single-molecule HiFi long reads was generated. Primary assembly contigs were scaffolded with chromosome conformation Hi-C data. Manual assembly curation corrected 6 missing joins or mis-joins and removed 2 haplotypic duplications, reducing the scaffold number by 13.51%.

**Figure 2.**
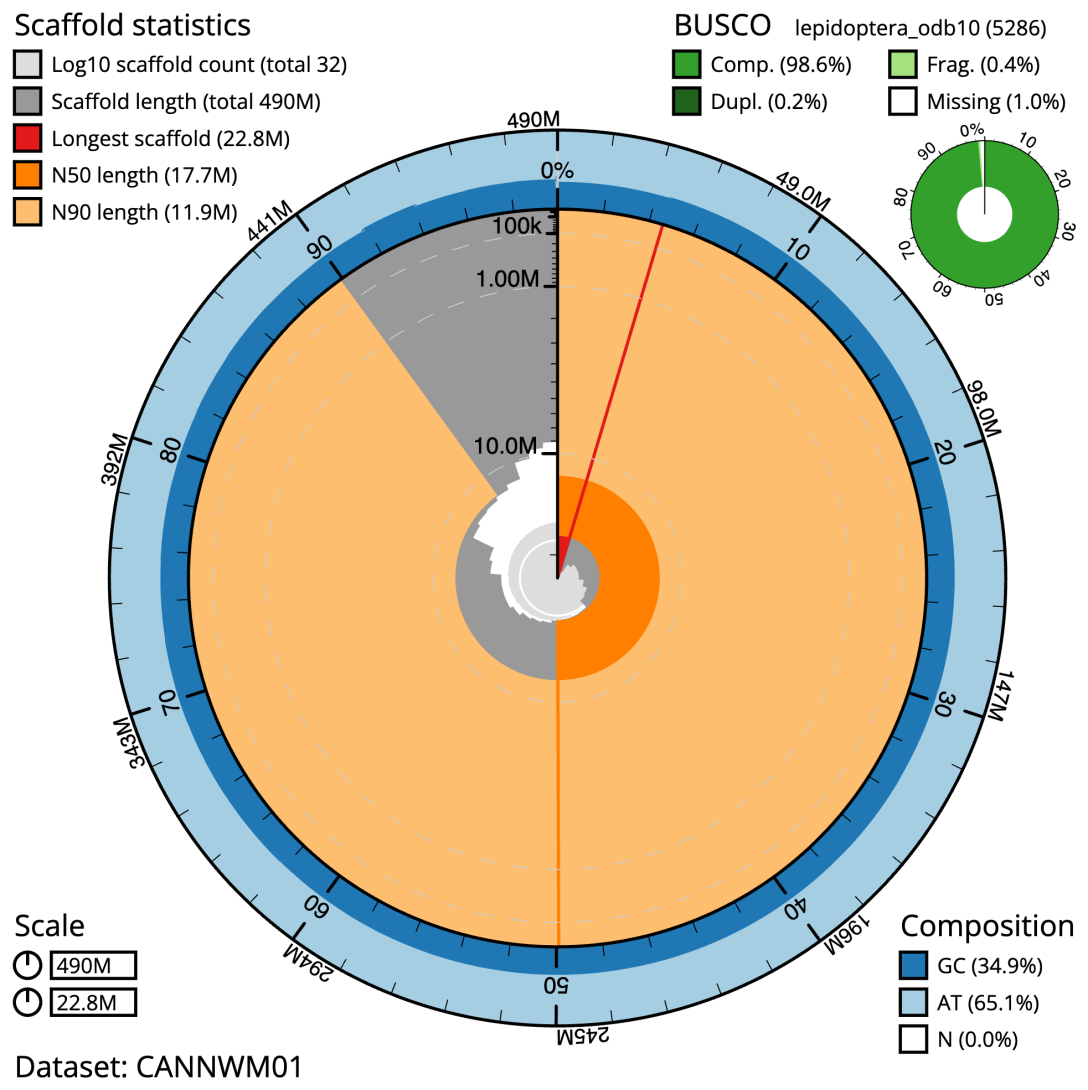
Genome assembly of *Saturnia pavonia*, ilSatPavo1.1: metrics. The BlobToolKit Snailplot shows N50 metrics and BUSCO gene completeness. The main plot is divided into 1,000 size-ordered bins around the circumference with each bin representing 0.1% of the 489,898,868 bp assembly. The distribution of scaffold lengths is shown in dark grey with the plot radius scaled to the longest scaffold present in the assembly (22,760,314 bp, shown in red). Orange and pale-orange arcs show the N50 and N90 scaffold lengths (17,680,408 and 11,900,431 bp), respectively. The pale grey spiral shows the cumulative scaffold count on a log scale with white scale lines showing successive orders of magnitude. The blue and pale-blue area around the outside of the plot shows the distribution of GC, AT and N percentages in the same bins as the inner plot. A summary of complete, fragmented, duplicated and missing BUSCO genes in the lepidoptera_odb10 set is shown in the top right. An interactive version of this figure is available at https://blobtoolkit.genomehubs.org/view/CANNWM01/dataset/CANNWM01/snail.

**Figure 3.**
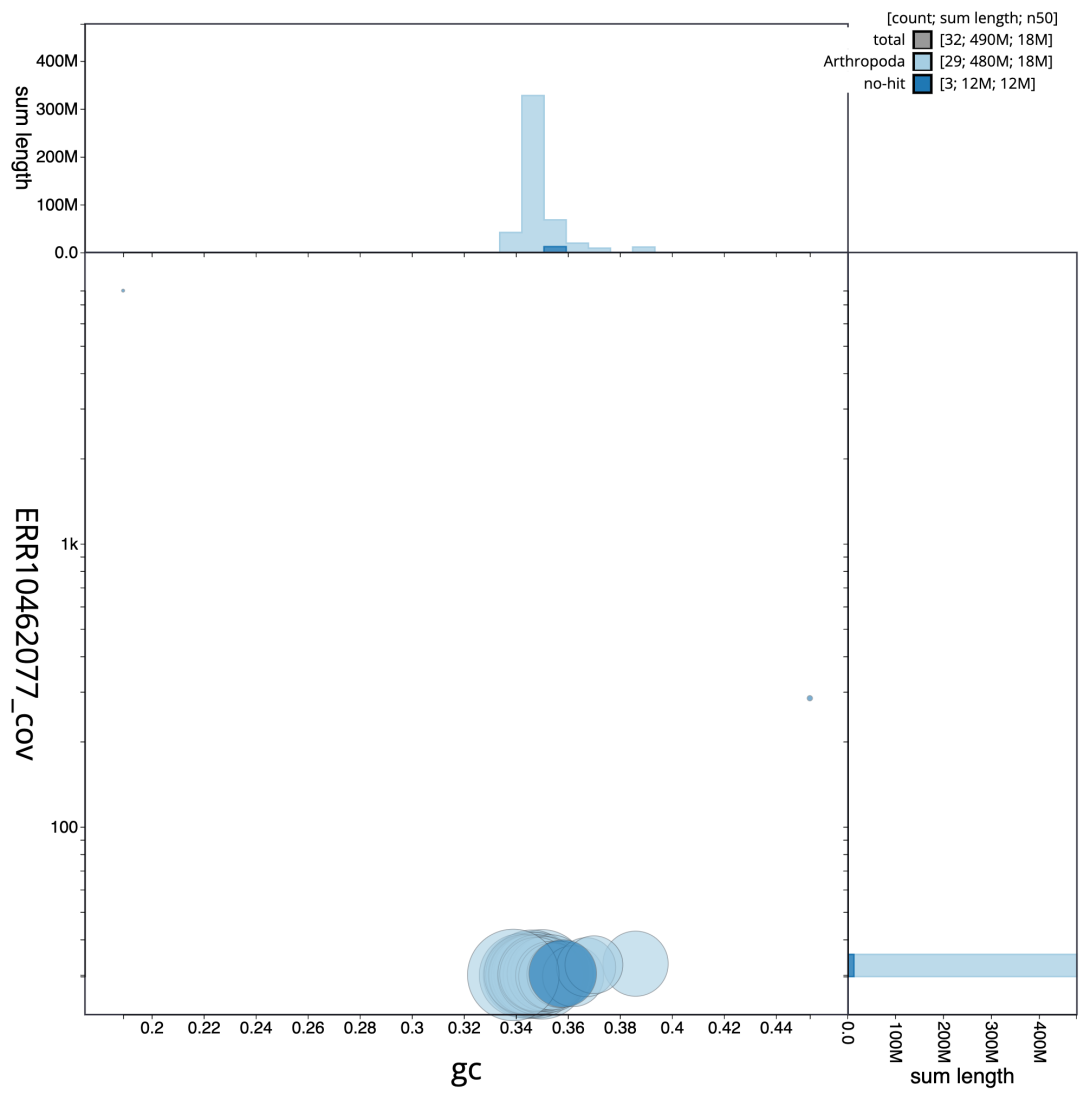
Genome assembly of *Saturnia pavonia*, ilSatPavo1.1: BlobToolKit GC-coverage plot. Scaffolds are coloured by phylum. Circles are sized in proportion to scaffold length. Histograms show the distribution of scaffold length sum along each axis. An interactive version of this figure is available at https://blobtoolkit.genomehubs.org/view/CANNWM01/dataset/CANNWM01/blob.

**Figure 4.**
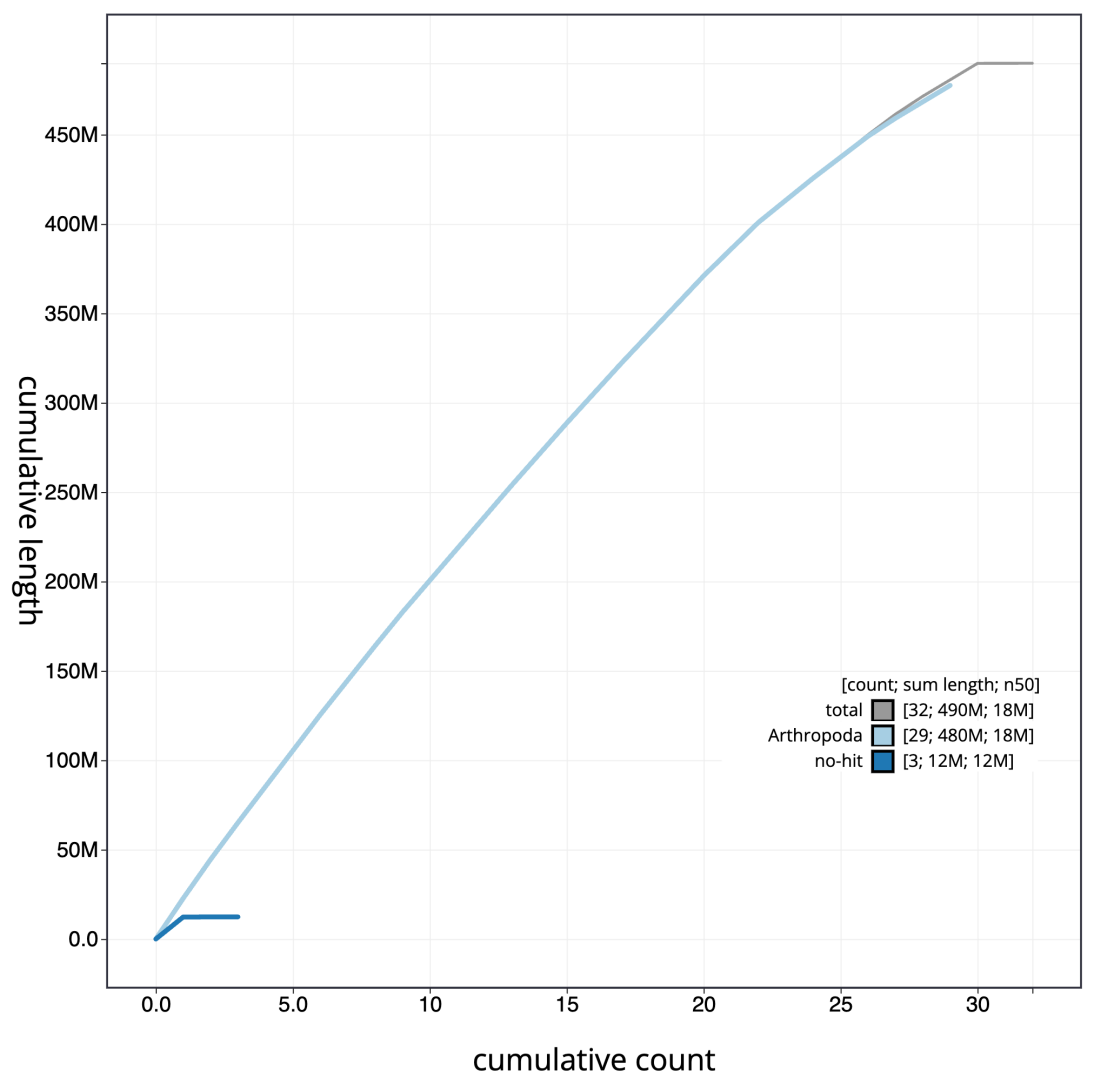
Genome assembly of *Saturnia pavonia*, ilSatPavo1.1: BlobToolKit cumulative sequence plot. The grey line shows cumulative length for all scaffolds. Coloured lines show cumulative lengths of scaffolds assigned to each phylum using the buscogenes taxrule. An interactive version of this figure is available at https://blobtoolkit.genomehubs.org/view/CANNWM01/dataset/CANNWM01/cumulative.

**Figure 5.**
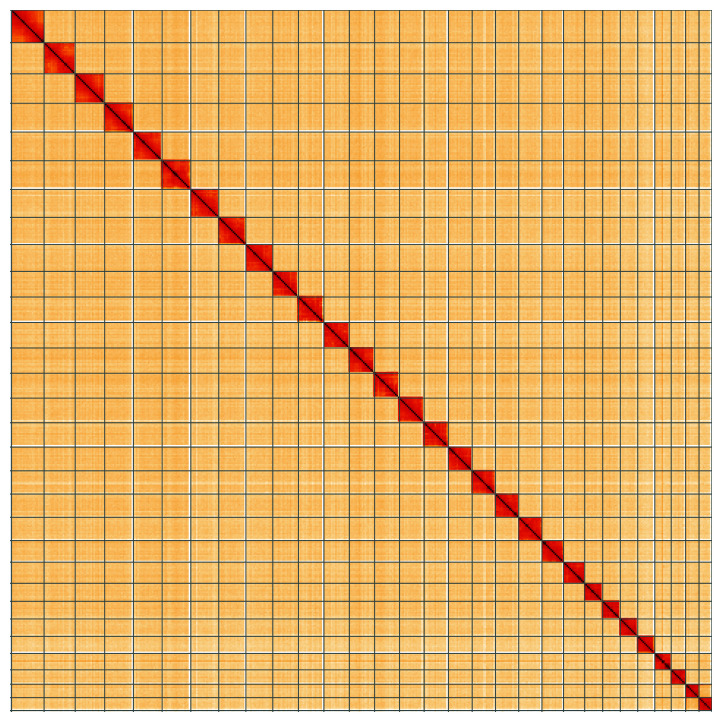
Genome assembly of *Saturnia pavonia*, ilSatPavo1.1: Hi-C contact map of the ilSatPavo1.1 assembly, visualised using HiGlass. Chromosomes are shown in order of size from left to right and top to bottom. An interactive version of this figure may be viewed at https://genome-note-higlass.tol.sanger.ac.uk/l/?d=SljOrrzkSG67snu-sBNiBg.

The final assembly has a total length of 489.9 Mb in 31 sequence scaffolds with a scaffold N50 of 17.7 Mb ( Table 1). The snailplot in Figure 2 provides a summary of the assembly statistics, while the distribution of assembly scaffolds on GC proportion and coverage is shown in Figure 3. The cumulative assembly plot in Figure 4 shows curves for subsets of scaffolds assigned to different phyla. Most (99.98%) of the assembly sequence was assigned to 30 chromosomal-level scaffolds, representing 29 autosomes and the Z sex chromosome. Chromosome-scale scaffolds confirmed by the Hi-C data are named in order of size ( Figure 5; Table 2). While not fully phased, the assembly deposited is of one haplotype. Contigs corresponding to the second haplotype have also been deposited. The mitochondrial genome was also assembled and can be found as a contig within the multifasta file of the genome submission.

**Table 1.** Genome data for *Saturnia pavonia*, ilSatPavo1.1.

Project accession data		
Assembly identifier	ilSatPavo1.1	
Species	*Saturnia pavonia*	
Specimen	ilSatPavo1	
NCBI taxonomy ID	332931	
BioProject	PRJEB57274	
BioSample ID	SAMEA110451586	
Isolate information	ilSatPavo1, male: thorax (DNA and Hi-C sequencing), abdomen (RNA sequencing)	
Assembly metrics *		*Benchmark*		
Consensus quality (QV)	68.6	*≥ 50*
*k*-mer completeness	100.0%	*≥ 95%*
BUSCO **	C:98.6%[S:98.4%,D:0.2%], F:0.4%,M:1.0%,n:5,286	*C ≥ 95%*
Percentage of assembly mapped to chromosomes	99.98%	*≥ 95%*
Sex chromosomes	Z	*localised homologous pairs*
Organelles	Mitochondrial genome: 15.29 kb	*complete single alleles*
Raw data accessions		
PacificBiosciences SEQUEL II	ERR10462077	
Hi-C Illumina	ERR10466811	
PolyA RNA-Seq Illumina	ERR11606290	
Genome assembly		
Assembly accession	GCA_947532125.1	
*Accession of alternate haplotype*	GCA_947532135.1	
Span (Mb)	489.9	
Number of contigs	72	
Contig N50 length (Mb)	13.2	
Number of scaffolds	31	
Scaffold N50 length (Mb)	17.7	
Longest scaffold (Mb)	22.76	
Genome annotation		
Number of protein-coding genes	11,903	
Number of non-coding genes	1,739	
Number of gene transcripts	22,289	

* Assembly metric benchmarks are adapted from column VGP-2020 of “Table 1: Proposed standards and metrics for defining genome assembly quality” from ( Rhie *et al.*, 2021). ** BUSCO scores based on the lepidoptera_odb10 BUSCO set using version 5.3.2. C = complete [S = single copy, D = duplicated], F = fragmented, M = missing, n = number of orthologues in comparison. A full set of BUSCO scores is available at https://blobtoolkit.genomehubs.org/view/CANNWM01/dataset/CANNWM01/busco.

**Table 2.** Chromosomal pseudomolecules in the genome assembly of *Saturnia pavonia*, ilSatPavo1.

INSDC accession	Chromosome	Length (Mb)	GC%
OX383895.1	1	21.77	35.0
OX383896.1	2	20.59	34.5
OX383897.1	3	20.11	34.5
OX383898.1	4	20.09	34.5
OX383899.1	5	20.08	34.5
OX383900.1	6	19.53	34.5
OX383901.1	7	18.97	34.0
OX383902.1	8	18.82	34.5
OX383903.1	9	17.89	34.5
OX383904.1	10	17.84	34.5
OX383905.1	11	17.8	34.5
OX383906.1	12	17.68	34.5
OX383907.1	13	17.4	34.5
OX383908.1	14	17.22	34.5
OX383909.1	15	17.0	34.5
OX383910.1	16	16.6	35.0
OX383911.1	17	16.28	35.0
OX383912.1	18	16.26	35.5
OX383913.1	19	16.23	35.0
OX383914.1	20	15.11	35.0
OX383915.1	21	14.8	35.0
OX383916.1	22	12.6	35.5
OX383917.1	23	12.32	36.0
OX383918.1	24	12.19	35.5
OX383919.1	25	11.9	35.5
OX383920.1	26	11.49	38.5
OX383921.1	27	10.06	36.0
OX383922.1	28	9.4	36.5
OX383923.1	29	8.99	37.0
OX383894.1	Z	22.76	34.0
OX383924.1	MT	0.02	19.0

The estimated Quality Value (QV) of the final assembly is 68.6 with *k*-mer completeness of 100.0%, and the assembly has a BUSCO v5.3.2 completeness of 98.6% (single = 98.4%, duplicated = 0.2%), using the lepidoptera_odb10 reference set ( *n* = 5,286).

Metadata for specimens, barcode results, spectra estimates, sequencing runs, contaminants and pre-curation assembly statistics are given at https://links.tol.sanger.ac.uk/species/332931.

## Genome annotation report

The *Saturnia pavonia* genome assembly (GCA_947532125.1) was annotated using the Ensembl rapid annotation pipeline ( Table 1; https://rapid.ensembl.org/Saturnia_pavonia_GCA_947532125.1/Info/Index). The resulting annotation includes 22,289 transcribed mRNAs from 11,903 protein-coding and 1,739 non-coding genes.

## Methods

### Sample acquisition and nucleic acid extraction

The *Saturnia pavonia* specimen used for genome sequencing and Hi-C data (specimen ID Ox002139, ToLID ilSatPavo1) was collected as a larva from Wytham Woods, Oxfordshire (biological vice-county Berkshire), UK (latitude 51.77, longitude –1.33) on 09/08/2021 by Ellen Baker (University of Oxford). The larva was reared by Liam Crowley (University of Oxford). The adult moth eclosed on 02/05/2022 and was preserved on dry ice.

Protocols developed by the Wellcome Sanger Institute (WSI) Tree of Life core laboratory have been published on protocols.io ( Denton *et al.*, 2023b). The workflow for high molecular weight (HMW) DNA extraction at the WSI includes a sequence of core procedures: sample preparation; sample homogenisation, DNA extraction, fragmentation, and clean-up. In sample preparation, the ilSatPavo1 sample was weighed and dissected on dry ice ( Jay *et al.*, 2023). Tissue from the thorax was homogenised using a PowerMasher II tissue disruptor ( Denton *et al.*, 2023a). HMW DNA was extracted in the WSI Scientific Operations core using the Automated MagAttract v2 protocol ( Oatley *et al.*, 2023). HMW DNA was sheared into an average fragment size of 12–20 kb in a Megaruptor 3 system with speed setting 31 ( Bates *et al.*, 2023). Sheared DNA was purified by solid-phase reversible immobilisation ( Strickland *et al.*, 2023): in brief, the method employs a 1.8X ratio of AMPure PB beads to sample to eliminate shorter fragments and concentrate the DNA. The concentration of the sheared and purified DNA was assessed using a Nanodrop spectrophotometer and Qubit Fluorometer and Qubit dsDNA High Sensitivity Assay kit. Fragment size distribution was evaluated by running the sample on the FemtoPulse system.

RNA was extracted from abdomen tissue of ilSatPavo1 in the Tree of Life Laboratory at the WSI using the RNA Extraction: Automated MagMax™ *mir*Vana protocol ( do Amaral *et al.*, 2023). The RNA concentration was assessed using a Nanodrop spectrophotometer and a Qubit Fluorometer using the Qubit RNA Broad-Range Assay kit. Analysis of the integrity of the RNA was done using the Agilent RNA 6000 Pico Kit and Eukaryotic Total RNA assay.

### Sequencing

Pacific Biosciences HiFi circular consensus DNA sequencing libraries were constructed according to the manufacturers’ instructions. Poly(A) RNA-Seq libraries were constructed using the NEB Ultra II RNA Library Prep kit. DNA and RNA sequencing was performed by the Scientific Operations core at the WSI on Pacific Biosciences SEQUEL II (HiFi) and Illumina NovaSeq 6000 (RNA-Seq) instruments. Hi-C data were also generated from remaining thorax tissue of ilSatPavo1 using the Arima2 kit and sequenced on the Illumina NovaSeq 6000 instrument.

### Genome assembly, curation and evaluation

Assembly was carried out with Hifiasm ( Cheng *et al.*, 2021) and haplotypic duplication was identified and removed with purge_dups ( Guan *et al.*, 2020). The assembly was then scaffolded with Hi-C data ( Rao *et al.*, 2014) using YaHS ( Zhou *et al.*, 2023). The assembly was checked for contamination and corrected using the gEVAL system ( Chow *et al.*, 2016) as described previously ( Howe *et al.*, 2021). Manual curation was performed using gEVAL, HiGlass ( Kerpedjiev *et al.*, 2018) and Pretext ( Harry, 2022). The mitochondrial genome was assembled using MitoHiFi ( Uliano-Silva *et al.*, 2023), which runs MitoFinder ( Allio *et al.*, 2020) or MITOS ( Bernt *et al.*, 2013) and uses these annotations to select the final mitochondrial contig and to ensure the general quality of the sequence.

A Hi-C map for the final assembly was produced using bwa-mem2 ( Vasimuddin *et al.*, 2019) in the Cooler file format ( Abdennur & Mirny, 2020). To assess the assembly metrics, the *k*-mer completeness and QV consensus quality values were calculated in Merqury ( Rhie *et al.*, 2020). This work was done using Nextflow ( Di Tommaso *et al.*, 2017) DSL2 pipelines “sanger-tol/readmapping” ( Surana *et al.*, 2023a) and “sanger-tol/genomenote” ( Surana *et al.*, 2023b). The genome was analysed within the BlobToolKit environment ( Challis *et al.*, 2020) and BUSCO scores ( Manni *et al.*, 2021; Simão *et al.*, 2015) were calculated.

Table 3 contains a list of relevant software tool versions and sources.

**Table 3.** Software tools: versions and sources.

Software tool	Version	Source
BlobToolKit	4.1.7	https://github.com/blobtoolkit/blobtoolkit
BUSCO	5.3.2	https://gitlab.com/ezlab/busco
Hifiasm	0.16.1-r375	https://github.com/chhylp123/hifiasm
HiGlass	1.11.6	https://github.com/higlass/higlass
Merqury	MerquryFK	https://github.com/thegenemyers/MERQURY.FK
MitoHiFi	2	https://github.com/marcelauliano/MitoHiFi
PretextView	0.2	https://github.com/wtsi-hpag/PretextView
purge_dups	1.2.3	https://github.com/dfguan/purge_dups
sanger-tol/genomenote	v1.0	https://github.com/sanger-tol/genomenote
sanger-tol/readmapping	1.1.0	https://github.com/sanger-tol/readmapping/tree/1.1.0
YaHS	1.1a.2	https://github.com/c-zhou/yahs

### Genome annotation

The Ensembl gene annotation system ( Aken *et al.*, 2016) was used to generate annotation for the *Saturnia pavonia* assembly (GCA_947532125.1). Annotation was created primarily through alignment of transcriptomic data to the genome, with gap filling via protein-to-genome alignments of a select set of proteins from UniProt ( UniProt Consortium, 2019).

### Wellcome Sanger Institute – Legal and Governance

The materials that have contributed to this genome note have been supplied by a Darwin Tree of Life Partner. The submission of materials by a Darwin Tree of Life Partner is subject to the **‘Darwin Tree of Life Project Sampling Code of Practice’**, which can be found in full on the Darwin Tree of Life website here. By agreeing with and signing up to the Sampling Code of Practice, the Darwin Tree of Life Partner agrees they will meet the legal and ethical requirements and standards set out within this document in respect of all samples acquired for, and supplied to, the Darwin Tree of Life Project. 

Further, the Wellcome Sanger Institute employs a process whereby due diligence is carried out proportionate to the nature of the materials themselves, and the circumstances under which they have been/are to be collected and provided for use. The purpose of this is to address and mitigate any potential legal and/or ethical implications of receipt and use of the materials as part of the research project, and to ensure that in doing so we align with best practice wherever possible. The overarching areas of consideration are:

•   Ethical review of provenance and sourcing of the material

•   Legality of collection, transfer and use (national and international) 

Each transfer of samples is further undertaken according to a Research Collaboration Agreement or Material Transfer Agreement entered into by the Darwin Tree of Life Partner, Genome Research Limited (operating as the Wellcome Sanger Institute), and in some circumstances other Darwin Tree of Life collaborators.

## Data Availability

European Nucleotide Archive: *Saturnia pavonia* (emperor moth). Accession number PRJEB57274; https://identifiers.org/ena.embl/PRJEB57274 ( Wellcome Sanger Institute, 2022). The genome sequence is released openly for reuse. The *Saturnia pavonia* genome sequencing initiative is part of the Darwin Tree of Life (DToL) project. All raw sequence data and the assembly have been deposited in INSDC databases. Raw data and assembly accession identifiers are reported in Table 1.

## Author information

Members of the University of Oxford and Wytham Woods Genome Acquisition Lab are listed here: https://doi.org/10.5281/zenodo.7125292.

Members of the Darwin Tree of Life Barcoding collective are listed here: https://doi.org/10.5281/zenodo.4893703.

Members of the Wellcome Sanger Institute Tree of Life Management, Samples and Laboratory team are listed here: https://doi.org/10.5281/zenodo.10066175.

Members of Wellcome Sanger Institute Scientific Operations: Sequencing Operations are listed here: https://doi.org/10.5281/zenodo.10043364.

Members of the Wellcome Sanger Institute Tree of Life Core Informatics team are listed here: https://doi.org/10.5281/zenodo.10066637.

Members of the Tree of Life Core Informatics collective are listed here: https://doi.org/10.5281/zenodo.5013541.

Members of the Darwin Tree of Life Consortium are listed here: https://doi.org/10.5281/zenodo.4783558.
