# Supplementary material for: The genome sequence of the Emperor moth, Saturnia pavonia (Linnaeus, 1758)
Source: Wellcome Open Res. 2024 Feb 19;9:48. [Version 1] doi: 10.12688/wellcomeopenres.20652.1 (PMC11101921; doi:10.12688/wellcomeopenres.20652.1)
Supplement: Peer review history

# Reviewer report: Reviewer response for version 1

**DOI:** 10.21956/wellcomeopenres.22857.r82138

**Published:** 2024-05-21

**Authors:** Marco Gerdol

**Affiliations:** 1 University of Trieste, Trieste, Italy

The manuscript by Crowley and colleagues reports the genome assembly of the Emperor moth, *Saturnia pavonia*. This resource was obtained as a part of the Darwin Tree of Life project, through the use of a highly standardized and reproducible pipeline. Therefore, there are no significant methodological concerns, and the quality of the assembled genome appears to be very high.

 I just have a very few minor comments dealing with cosmetic changes that the authors may consider including in a revised version.

 “(51.77, –1.33)” -> I am not sure this is the standard or most common way of reporting geographical coordinates. Please add “latitude” and “longitude”.

 Genome annotation report: this was obtain though the Ensembl rapid annotation pipeline, which in other phyla (I am not really sure about arthropods) performs rather poorly. Do the BUSCO metrics obtained by analyzing the annotated gene set match those reported in the previous paragraph by analyzing the genome assembly?

 Data availability: consider adding here the link to the Ensembl rapid annotation report.

Are sufficient details of methods and materials provided to allow replication by others?

Yes

Is the rationale for creating the dataset(s) clearly described?

Yes

Are the datasets clearly presented in a useable and accessible format?

Yes

Are the protocols appropriate and is the work technically sound?

Yes

Reviewer Expertise:

non-model species genomics

I confirm that I have read this submission and believe that I have an appropriate level of expertise to confirm that it is of an acceptable scientific standard.

# Reviewer report: Reviewer response for version 1

**DOI:** 10.21956/wellcomeopenres.22857.r78300

**Published:** 2024-05-17

**Authors:** Peter Dearden

**Affiliations:** 1 University of Otago, Dunedin, Otago, New Zealand

This report describes the genome sequencing of a large day-flying moth; the Emperor moth ( *Saturnia pavonia*). The report describes the key biological details of the moth well, including the relationships between the sampled British populations, and those in the rest of Europe. Photos are provided of the sampled animal from both larval and adult stages.

 The genome report indicates that this is a very high-quality, contiguous, uncontaminated genome. the BUSCO scores show the genome is highly complete, and indicates the high quality of the assembly. 

 The data generated is available, and the tools used to carry out the work, are well described and available to others.

 I strongly support this report. I would ask that in the section where the authors are describing the correction of scaffolds that they state the numbers of scaffolds affected, rather than just a percentage.

Are sufficient details of methods and materials provided to allow replication by others?

Yes

Is the rationale for creating the dataset(s) clearly described?

Yes

Are the datasets clearly presented in a useable and accessible format?

Yes

Are the protocols appropriate and is the work technically sound?

Yes

Reviewer Expertise:

Insect Genomics

I confirm that I have read this submission and believe that I have an appropriate level of expertise to confirm that it is of an acceptable scientific standard.

# Reviewer report: Reviewer response for version 1

**DOI:** 10.21956/wellcomeopenres.22857.r82144

**Published:** 2024-05-17

**Authors:** Jaakko Pohjoismäki

**Affiliations:** 1 University of Eastern Finland, Joensuu, Finland

The genome report by Liam M Crowley, Ellen Baker, Peter WH Holland and the DToL consortium presents the reference genome assembly of the Emperor moth. It is a nice addition of an iconic species to the growing number of Lepidopteran genomes from the DToL.

 Background

 The background is well written and concise. One could add that *pavonia* is replaced by *pavoniella* in the east and that the two species have been separated only recently. This close relatedness might then deserve a comment regarding the utility of the genome assembly? Also, the pavonia is fairly early spring species, so I would imagine that May is too late for the flight time in the UK? As of note, there are many old studies on the pheromone attracting marked males, but these will require a bit of digging as they are difficult to find. Main point is that not all estimates are based on "conjecture".

 The larvae are also sexually dimorphic, but this is maybe an unnecessary detail.

 The genome assembly, annotation and completeness looks very good, representing the DToL quality standards. The methods are adequately described and following the DToL genome production pipeline without any issues.

 My main complaint is that the genome assembly is from a male and thus lacks the W sex chromosome. I think this is a missed opportunity to collect valuable genome data on the sex chromosome evolution among insects. For *Saturnia pavonia* missing this opportunity is especially unnecessary, as the females are easily available from light traps, collecting as larvae or bought from a (British) web shop ( https://www.wwb.co.uk/emperor-moth-pavonia-cocoons).

 However, this detail does not otherwise affect the quality of the work. So far the use of males for the genome assemblies has been a recurrent issue with the Lepidopteran genomes. I hope this will be avoided in the future and females will be used for the coming species.

Are sufficient details of methods and materials provided to allow replication by others?

Yes

Is the rationale for creating the dataset(s) clearly described?

Yes

Are the datasets clearly presented in a useable and accessible format?

Yes

Are the protocols appropriate and is the work technically sound?

Yes

Reviewer Expertise:

taxonomy; genetics; genomics; insects; mammals; molecular biology

I confirm that I have read this submission and believe that I have an appropriate level of expertise to confirm that it is of an acceptable scientific standard, however I have significant reservations, as outlined above.

# Reviewer report: Reviewer response for version 1

**DOI:** 10.21956/wellcomeopenres.22857.r80533

**Published:** 2024-05-10

**Authors:** Christopher Cunningham

**Affiliations:** 1 University of Georgia, Athens, Georgia, USA

I think this is a good general resource for the genomics community. The methods are sound and relatively well documented. The article needs a few more details to be reasonably reproducible.

 Abstract. There is no justification within the abstract, just results. At least a sentence that this was part of a large survey of insects is needed. And one sentence for its possible value.

 Keywords. Not present.  

 Background. There is little true motivation for this study in the introduction. Nothing wrong is said, but the reader has little idea why the work was done. It’s perfectly fine that the work was done in the context of a large survey, but tell the reader that. Almost all the details of its life history are irrelevant to the reader. The paper is not about its ecology or morphology, but about its genetics. That is not mentioned once.

             The end paragraph mentions it is an existing or potential model for chemical defenses and pheromone communication but not direct justification or citation of why this species is particularly useful for these is given.

 Figure 2/3/4/5 titles. These should be informative and tell the reader what you would like them to understand about the data; e.g., Fig 3. Little contamination was found in the final genome assembly.

 Figure 4. has very little information and can easily be summarized as a sentence in the results. The same is true of Fig 5

 All parameter setting if they were different from default of each piece of bioinformatic software needs to be specified. Just put a sentence to begin that everything was default unless specified otherwise. What assessment was done to ensure that default was adequate?

 A reasonable amount of genes is predicted. However, the reader has very little understanding of how these were evaluated beyond their initial generation. What QC beyond an automated annotation was done? One annotation run with default parameters should come with a strong disclaimer that any further work on specific gene families requires QC on the part of the user because the current annotation is a guide only.

             What is the BUSCO percentage of the predicted gene set? That is an absolute minimum for a reader to be able to assess the minimum quality of the annotation.

 Table 3 is of little to no value and contains much duplicated information. Just add the version numbers of software used inline at the appropriate places in the methods section.

 Is the Ethics statement needed? It just says the provider of the sample should meet some agreed upon standard, but does not actually say that they did in this case. Either drop the statement or actually say the standard was met for this sample. This is not a paper about the standards of collection.

 Add something to the report that contextualize the research for the reader. Is this species the first of a taxon to be assembled, is it an outgroup to some established model species, is it now possible to investigate some interesting aspect of the species biology, etc? This does not to be extensive or highly directed, but there is currently no justification of this work outside of the Data Availability Statement. Even Data Note should contain rationale for the work.

Are sufficient details of methods and materials provided to allow replication by others?

Partly

Is the rationale for creating the dataset(s) clearly described?

Partly

Are the datasets clearly presented in a useable and accessible format?

Yes

Are the protocols appropriate and is the work technically sound?

Yes

Reviewer Expertise:

Genomics, genetics, epigenetics, behavior, reproduction

I confirm that I have read this submission and believe that I have an appropriate level of expertise to confirm that it is of an acceptable scientific standard, however I have significant reservations, as outlined above.
